# Importance of *aggR* sequence variants detection for accurate molecular diagnosis of enteroaggregative *Escherichia coli*

**DOI:** 10.1128/spectrum.01441-25

**Published:** 2025-09-24

**Authors:** Alejandra M. G. Del Carpio, Claudia A. Freire, Tânia A. T. Gomes, Cecilia M. Abe, Waldir P. Elias

**Affiliations:** 1 Laboratório de Bacteriologia, Instituto Butantan 196591, São Paulo, Brazil; 2 Departamento de Microbiologia, Imunologia e Parasitologia, Escola Paulista de Medicina, Universidade Federal de São Paulo 28105, São Paulo, Brazil; University of Georgia College of Veterinary Medicine, Athens, Georgia, USA

**Keywords:** enteroaggregative *Escherichia coli*, EAEC, diagnosis, triplex-PCR, diarrhea

## Abstract

Enteroaggregative *Escherichia coli* (EAEC) is one of the main diarrhea causative agents. This *E. coli* pathotype is defined by an aggregative adherence pattern observed in HEp-2 cells. Molecular diagnosis is still a challenge due to EAEC genetic heterogeneity. Different publications described primers for *aggR* and *aatA* detection to characterize EAEC. Recently, *afpR* has been suggested as EAEC molecular marker of some strains, demonstrating the existence of two subpopulations within this pathotype: typical and atypical EAEC, respectively, comprising *aatA*+/*aggR*+ and *aatA*+/*afpR*+. Studies performed by our group, however, detected conflicting results using different primer pairs for *aggR*. Therefore, this study mainly aimed to analyze the variation of *aggR* sequences and its implication in EAEC characterization, defining consensus primers. As a consequence, a triplex-PCR targeting *aggR*, *aatA,* and *afpR* is proposed as a one-step molecular diagnosis for both typical and atypical EAEC. Previously reported primer sequences used for *aggR, aatA*, and *afpR* detection, and their annealing sites were analyzed using sequence databases. The best primers for each target were selected, and a triplex-PCR was standardized and validated. Database alignment of *aggR* revealed numerous variant regions along its sequence, impairing the annealing of most reported primers, while significant variations in the annealing sites of *aatA* and *afpR* primers were not observed. Broad-range primers for *aggR* were determined and combined with *aatA* and *afpR*, resulting in a triplex-PCR. Our triplex-PCR targeting *aggR, aatA*, and *afpR* successfully detected only EAEC, covering all *aggR* variants, promoting a broad and specific detection of this pathotype.

**IMPORTANCE:**

Phenotypical and genotypical characterization of enteroaggregative *Escherichia coli* (EAEC) is either time-consuming or of variable sensitivity. Therefore, developing an accurate molecular diagnostic method for this *E. coli* pathotype is relevant for public health. This study analyzed the primers currently used to detect typical EAEC-related genes (*aatA* and *aggR*), and *afpR* as a marker for atypical EAEC. After an extensive literature review, it was observed that the *aatA* primers are suitable for its broad detection. On the other hand, none of the *aggR* primer pairs described were able to detect all variants of the *aggR* gene. To address this issue, universal primers for *aggR* were proposed to improve the detection of all *aggR* variants. Considering the demand for a fast and accurate molecular approach for EAEC diagnosis, a triplex-PCR assay was developed, validated, and optimized using the universal *aggR* primers in combination with primers for *aatA* and *afpR*, detecting both typical and atypical EAEC.

## INTRODUCTION

Enteroaggregative *Escherichia coli* (EAEC) is among the most prevalent agents of diarrhea in both children and adults worldwide, and is also associated with travelers’ diarrhea and numerous diarrhea outbreaks (1–3). Children in low-income countries asymptomatically colonized by EAEC are more likely to experience reduced vaccine response, stunted growth, and cognitive impairments (4, 5). In the last years, EAEC has also been isolated from cases of bloodstream and urinary tract infections (1, 6–8).

This diarrheagenic *E. coli* pathotype described by Nataro et al. (9) was initially characterized by an aggregative adherence (AA) pattern observed in HEp-2 cell assays, where bacteria adhered to both cells and coverslip in a stacked bricks-like pattern. This characteristic has been considered the gold standard for EAEC identification, despite being time-consuming and requiring specialized laboratory structure and trained personnel (10). In this context, molecular methods arise as important diagnostic tools. Different EAEC genetic markers have been targeted in epidemiological studies (2, 11–14). The CVD432 probe was the first molecular tool described and used for EAEC identification (15). Over time, other genes emerged to broadly detect EAEC by PCR, using both chromosome-encoded (*aaiC* or *aaiG*) and/or plasmid-borne genes (*aggR, aatA,* or *aap*) (2, 3, 10, 11, 16–20). Except for *aaiC* and *aaiG*, which are part of a Type 6 secretion system (T6SS), *aggR, aatA*, and *aap* are located in a virulence plasmid named pAA that also harbors the genes responsible for the biogenesis of the aggregative adherence fimbriae (AAF), specific EAEC structures (1, 4, 15, 21). Currently, it is known that *aatA* (previously known as the CVD432 probe) is part of an operon that encodes a Type 1 secretion system (T1SS) (15, 22, 23); *aggR* encodes a transcriptional regulator of EAEC virulence genes (21); and *aap* encodes the antiaggregation protein named dispersin (22). The term atypical EAEC (aEAEC) was proposed to include some EAEC strains exhibiting the AA pattern but lacking *aggR* sequence, suggesting the absence of pAA, whereas typical EAEC (tEAEC) includes those EAEC harboring *aggR* (1, 4).

Recently, the AA-related plasmid (pAFP) was identified in Shiga toxin-producing EAEC strains harboring *aap* and *aatA* but lacking *aggR*. Instead, they harbor *afpR*, an AraC-type regulator of the aggregate-forming pili (AFP) operon (24, 25). Following the initial reports of AFP in hybrid strains, further genome analyses revealed the presence of the AFP operon in EAEC strains isolated from fecal samples (6, 25).

Initial epidemiological studies performed by our group (12, 13) used the CVD432 probe to identify EAEC strains. In more recent studies (10, 17, 26), EAEC strains have been identified by PCR to detect the *aatA* and *aggR* genetic markers. In our routine, some strains primarily identified as EAEC using the CVD432 presented intriguingly different results when submitted to PCR for *aggR* detection using different primer pairs described in the literature. These results strongly suggested the presence of sequence variation in *aggR*.

In order to clarify this observation, the present work carried out an extensive literature-based search targeting published primers for molecular detection of EAEC. Broad-range primers for *aggR* were determined and combined with *aatA* and *afpR* primers, developing a Triplex-PCR for the detection of both typical and atypical EAEC.

## MATERIALS AND METHODS

### Primer selection and database construction

A literature-based search targeting published primers for molecular detection of *aggR, aatA*, and *afpR* was performed in November 2022. The terms “*aggR*,” “*aatA*,” “*afpR,*” “transcriptional regulator enteroaggregative *Escherichia coli*,” “pCVD432,” and “aggregative forming pilus” were searched in the PubMed database (https://pubmed.ncbi.nlm.nih.gov/), resulting in 1,578 studies, which were reviewed for the description of oligonucleotides used. From this search, nine primer pairs were identified as recurrent, and the original articles describing them were selected to serve as the basis for our subsequent analysis.

In parallel, *E. coli* genomes available in the NCBI database (https://www.ncbi.nlm.nih.gov/) in the FASTA format were analyzed, focusing on the identification of the *aggR*, *aatA*, and *afpR* genes. A total of 257 genome sequences containing one of these EAEC markers were selected for the construction of the database. As a result, 508 complete nucleotide sequences were selected (224 *aggR*, 251 *aatA*, and 33 *afpR*). Three distinct databases (one for each target gene) were constructed using Sublime Text 4 software (https://www.sublimetext.com/). These databases are publicly available on the Butantan Institute Repository (https://repositorio.butantan.gov.br/handle/butantan/13765).

### Multiple alignments, primer analyses, and selection

A multiple alignment was performed with the Geneious Prime 2023.2 software, using the MUSCLE algorithm, to identify conserved and variable regions among the sequences of each database. The generated alignments were used to determine annealing sites within the conserved regions for the forward and reverse primers designed to detect *aggR*, *aatA*, and *afpR*. Differences in the percentage of *aggR* detection between all previously published primers and the respective candidate primers were assessed in the *aggR* database using the PCR Products tool available online on the Sequence Manipulation Suite (version 2) (https://www.bioinformatics.org/sms2/pcr_products.html). Alternative primers for *afpR* detection were designed based on its corresponding database multiple alignment, using the Geneious Prime 2023.2 software.

### Selection of *aggR*, *aatA*, and *afpR* primer candidates

Primer candidates for *aggR*, *aatA*, and *afpR* detection are described in Table 1. Amplicon sizes were calculated *in silico*, considering the annealing sites of the selected primer pairs within the reference genomes available on the NCBI database: *aggR* and *aatA* sequences of EAEC 042, the EAEC prototype strain isolated from feces (21) - accession number NC_017627.1; and *afpR* sequence of UPEC-46 (25) - accession number NZ_JAHBCK010000003.1, which is an EAEC isolated from urinary tract infection (EAEC/UPEC hybrid strain). Template DNA was prepared by boiling a colony from the respective culture on Lysogeny Broth (LB) agar in 200 µL of water for 10 min.

**TABLE 1:** Primers and conditions defined for single reactions and the proposed triplex-PCR for EAEC detection

Primers			
Target gene	Sequence (5′→3′)	Reference	Amplicon size (bp)
*aatA*	F: CTGGCGAAAGACTGTATCAT	(23)	630
	R: CAATGTATAGAAATCCGCTGTT		
*aggR*	F: CTAATTGTACAATCGATGTA	(21)	569
	R: TGCTTTGCTCATTCTTGATTGC	(27)	
*afpR*	F: CAAAAGTGTCCGCAAACATA	This study	386
	R: TAATCGCCAGCGTTTACTTA		

Single reactions	
Reagents	Reaction
Primers: 2.5 pmol of each	94°C for 5 min (1 cycle) 94°C for 1min (30 cycles) 53°C for 1min (30 cycles) 72°C for 1min (30 cycles) 72°C for 7 min (1 cycle)
dATP, dTTP, dCTP, and dGTP: 0.1 mM of each	
Taq DNA polymerase (Invitrogen): 1.5 U	
10× PCR buffer (Invitrogen): 2.5 µL	
MgCl_2_:2 mM	
DNA template: 1.0 µL	
Total volume: 25 µL	

Triplex-PCR	
Reagents	Reaction
Primers: 0.5 pmol of each	94°C for 5 min (1 cycle) 94°C for 1min (35 cycles) 53°C for 1min (35 cycles) 72°C for 1min (35 cycles) 72°C for 7 min (1 cycle)
dATP, dTTP, dCTP, and dGTP: 0.4 mM of each	
Taq DNA polymerase (Invitrogen): 3 U	
10× PCR buffer (Invitrogen): 5.0 µL	
MgCl_2_: 2 mM	
DNA template: 5.0 µL	
Total volume: 50 µL	

Single reactions were performed as described in Table 1, and PCR products were analyzed by agarose gel electrophoresis (1%). Strains used as controls are described in Table S1. A DNA-free reaction was also used as a negative control. The *aggR* and *afpR* amplicons were purified with Monarch PCR & DNA Cleanup Kit (New England Biolabs), according to the manufacturer’s instructions, and Sanger sequenced at the Human Genome and Stem Cell Research Center of the University of São Paulo. The obtained sequences were analyzed using BLAST (https://blast.ncbi.nlm.nih.gov/), setting the above-mentioned *aggR* and *afpR* sequences as references.

### Triplex-PCR for EAEC detection

After individually validating the selected primers for *aggR, aatA*, and *afpR* with control strains, a triplex-PCR targeting these three genes was standardized. DNA was obtained as mentioned above, and a pool containing both EAEC 042 and UPEC-46 DNA was used as a positive control. The conditions defined for the triplex-PCR protocol are provided in Table 1.

### Validation of the triplex-PCR for EAEC detection

The specificity of the triplex-PCR was firstly assessed by testing five pathogenic *E. coli* prototype strains and *E. coli* DH5α, as described in Table S1. In parallel, 24 *E. coli* strains that presented the AA pattern on HEp-2 cells but were molecularly characterized as typical enteropathogenic *E. coli* (tEPEC), atypical EPEC (aEPEC), or Shiga toxin-producing *E. coli* (STEC), were also tested (Table S2). The triplex-PCR specificity was verified by testing 163 *aatA*-positive EAEC isolates from epidemiological studies of the etiology of acute diarrhea in children (12, 13) (Table S2).

The results obtained by the triplex-PCR were further complemented by the search for the presence of *aggA*, *aafA*, *agg3A*, *agg4A*, and *agg5A* (pilin-encoding genes of the five AAF variants), *afpA1* (pilin-encoding gene of AFP) as well as *cseA* (the pilin-encoding gene of CS22), also assessed by PCR (Table S3) in 53 *aatA*-positive EAEC strains (13). The remaining 110 strains had already been evaluated for the presence of these genes in a previous study (17).

## RESULTS

### Selection and analyses of primers for *aggR*, *aatA*, and *afpR* detection

**Fig 1:**
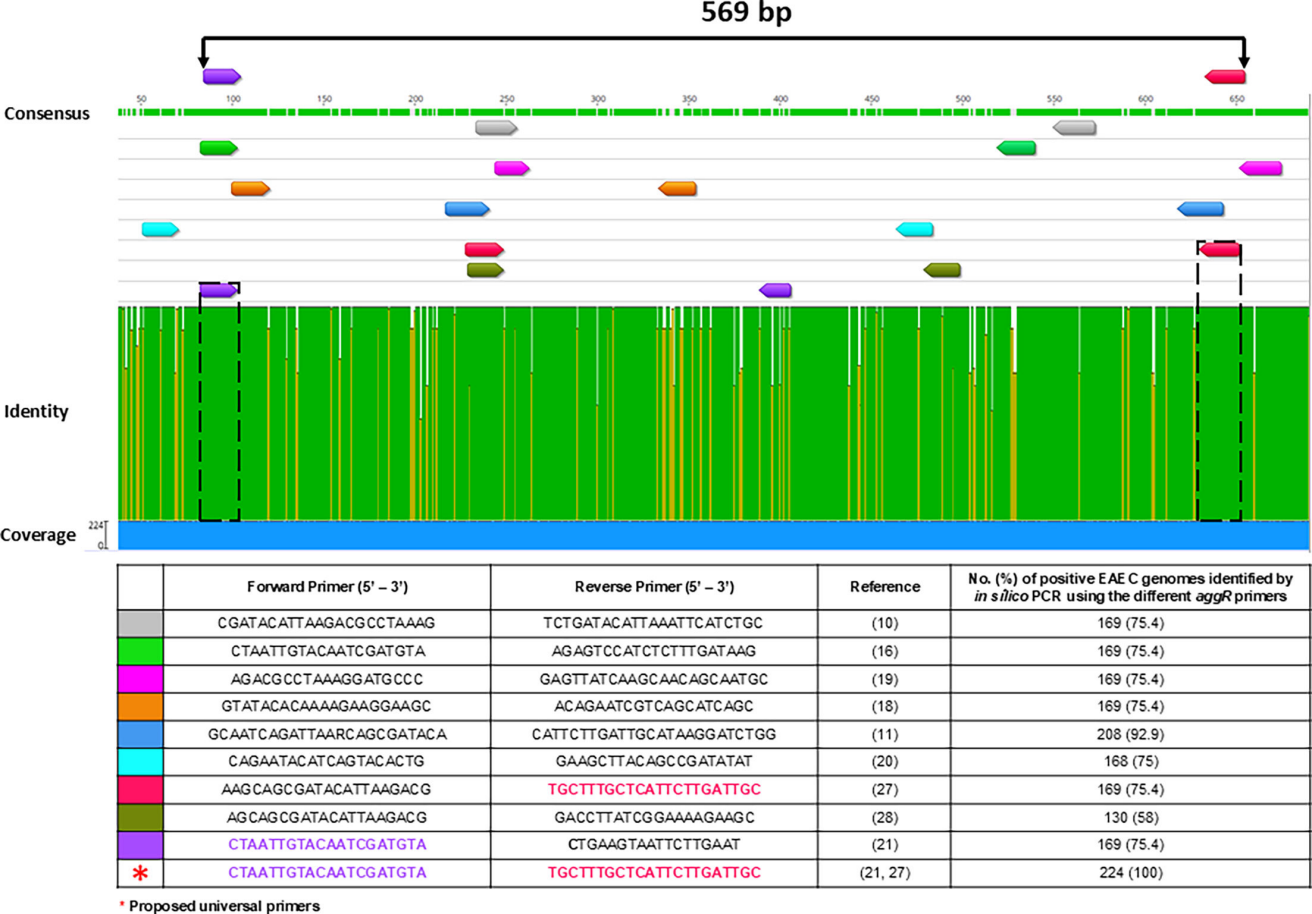
Alignment of *aggR* sequences indicating the annealing sites of published primers designed to detect the sequence. Conserved (green) and variable (yellow and white) regions were determined by aligning 224 *aggR* sequences downloaded from GenBank, using the MUSCLE algorithm available in Geneious software (version 2023.2.1). Arrows indicate the annealing sites of nine published primer pairs. The percentage of *aggR* detection using each listed primer pair by *in silico* PCR is also indicated (28).

**Fig 2:**
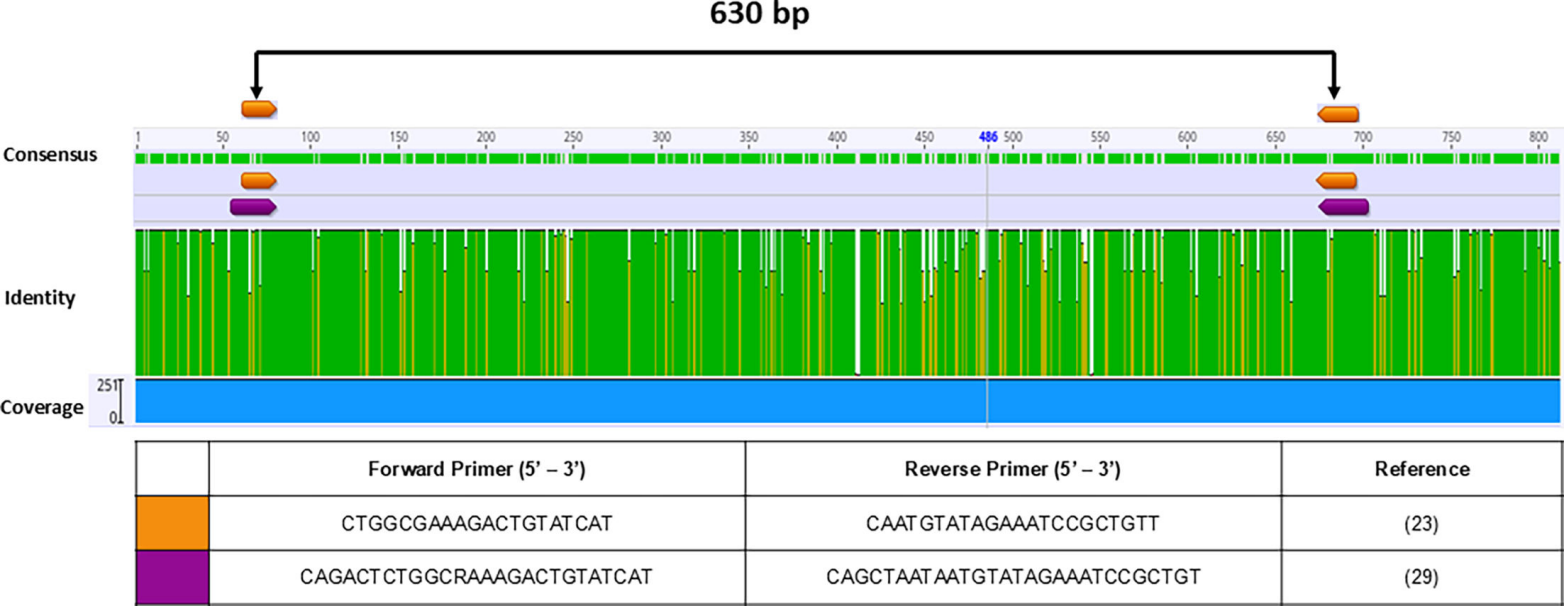
Alignment of *aatA* sequences indicating the annealing sites of published primers designed to detect the sequence. Conserved (green) and variable (yellow and white) regions were determined by aligning 251 *aatA* sequences downloaded from GenBank, using the MUSCLE algorithm available in Geneious software (version 2023.2.1). Arrows indicate the annealing sites of two published primer pairs.

**Fig 3:**
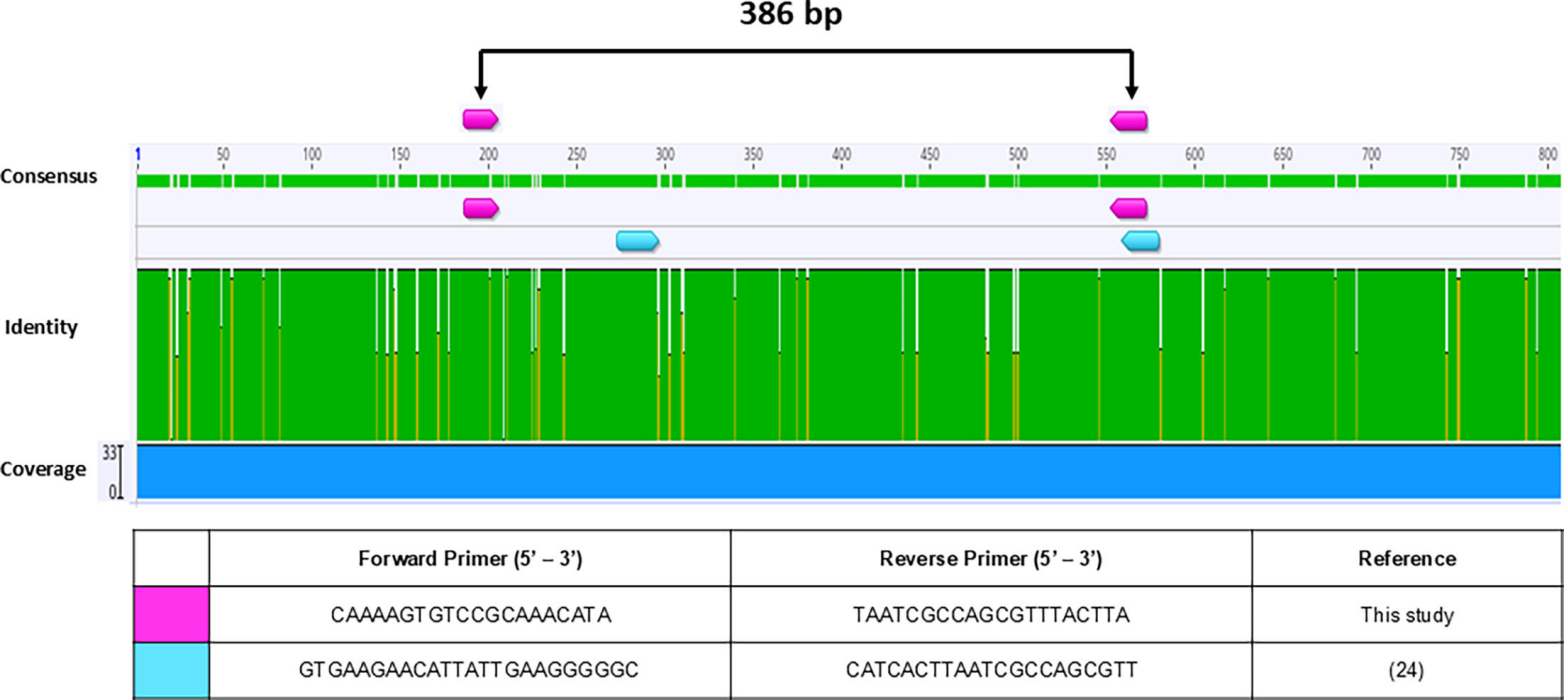
Alignment of *afpR* sequences indicating the annealing sites of published primers designed to detect the sequence**.** Conserved (green) and variable (yellow and white) regions were determined by aligning 33 *afpR* sequences downloaded from GenBank, using the MUSCLE algorithm available in Geneious software (version 2023.2.1). Arrows indicate the annealing sites of two published primer pairs.

A literature-based search provided different numbers of primers for each targeted gene (Fig. 1 to 3). The search for *aggR* primers resulted in nine original pairs (10, 11, 16, 18–21, 27, 28). The multiple alignments of the 224 *aggR* sequences revealed that sequence variations occur throughout the gene sequence, and most of the analyzed primer sequences anneal into these regions (Fig. 1). Only one forward and one reverse sequence, respectively proposed by Czeczulin et al. (21) and Trung et al. (27), annealed in conserved sites and were thus selected as a universal primer pair for *aggR* detection.

Such diversity of *aggR* primers with variable annealing regions suggested that some EAEC strains could present different results for *aggR* detection. For this reason, we compared the percentage of positivity in *aggR* detection between the previously published and the universal primers (Fig. 1). *In silico* analysis of primers described in the literature detected *aggR* in 58%–92.9% of the 224 strains analyzed, whereas the universal primers used in this study detected it in 100%, demonstrating excellent diagnostic performance.

Two original primer pairs were found for *aatA* detection (23, 29). Figure 2 shows *aatA* primers’ annealing sites, using the multiple alignments obtained with the 251-sequence database. The forward and reverse sequences of both sets anneal in overlapping regions of *aatA* with a few nucleotide variations between the sequences analyzed, indicating that both pairs are suitable for broad detection of *aatA* (Fig. S1). The first described primer pair proposed by Schmidt et al. (23) was chosen to integrate the triplex-PCR.

Concerning *afpR*, the primers proposed by Lang et al. (24) were the only ones described in the literature (Fig. 3). Although the reverse sequence anneals to a completely conserved site, a single nucleotide corresponding to the 3′ end of the forward sequence anneals to a variation point. Initially, the forward primer was adapted by excluding this 3′ end nucleotide. However, the attempts to standardize the triplex-PCR protocol using the adapted sequence failed, and a new primer pair for *afpR* detection was designed for this purpose (Fig. 3).

After single PCRs, the resulting *aggR* and *afpR* amplicons were purified and sequenced. The correct alignment of the amplicons with the corresponding *aggR* (EAEC 042) and *afpR* (UPEC-46) sequences observed in Fig. S2 and S3 enabled the proposal of a triplex-PCR for broad EAEC detection (Table 1).

### Standardization of a triplex-PCR for EAEC detection

**Fig 4:**
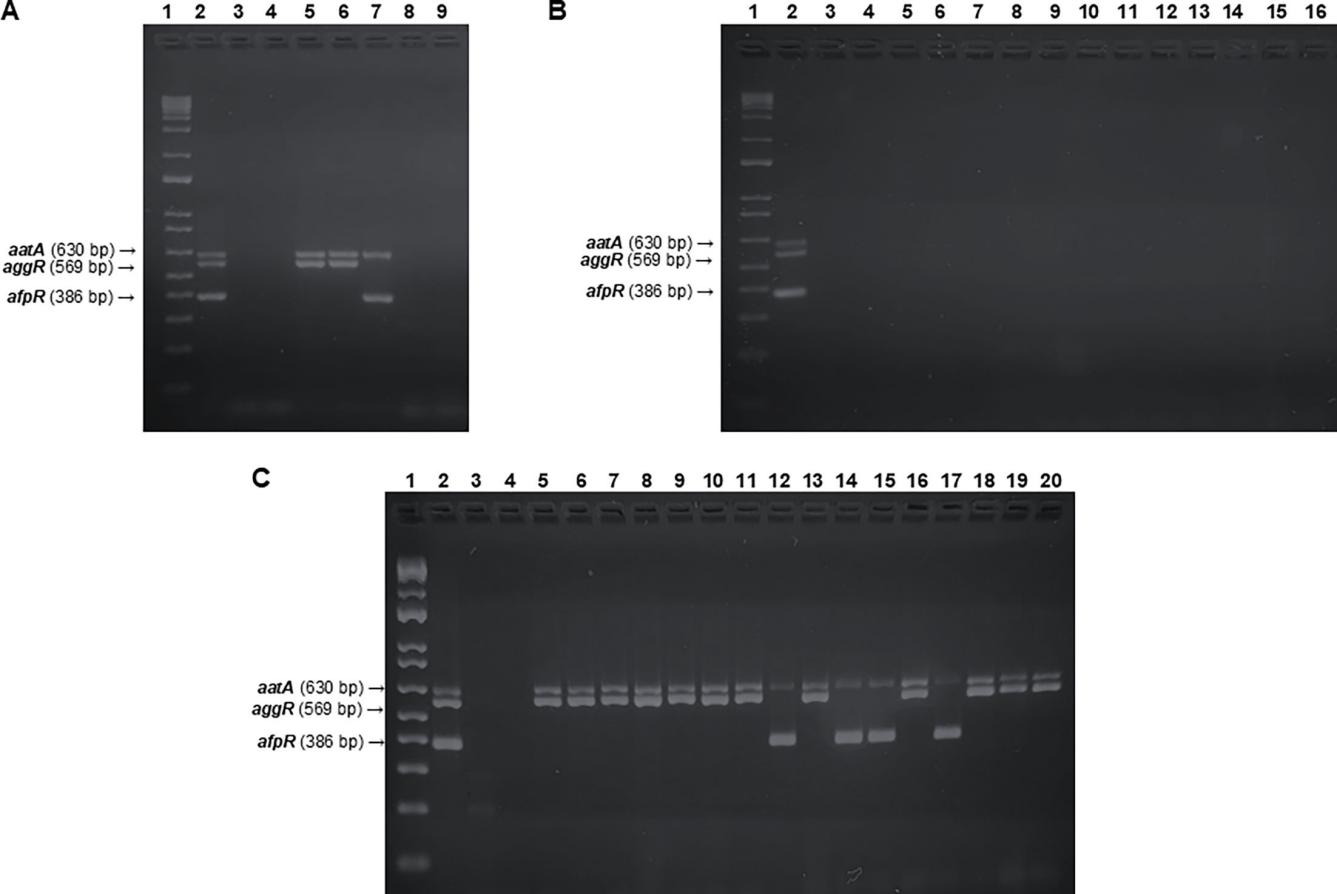
Standardization and validation of the triplex-PCR-based protocol for EAEC detection. Analyses of amplification products by electrophoresis in 2.0% agarose gels, stained with Unisafe dye (Uniscience). (**A**) Lane 1, 1 kb Plus DNA Ladder (Invitrogen); Lane 2, positive control - pool containing both EAEC 042 and UPEC-46 DNA; Lane 3, negative control using *E. coli* DH5α; Lane 4, negative control without DNA; Lane 5, EAEC 042; Lane 6, EAEC 17-2; Lane 7, UPEC-46; Lane 8, EPEC E2348/69; Lane 9, UPEC CFT073. (**B**) Lane 1, 1 kb Plus DNA Ladder (Invitrogen); Lane 2, positive control - pool containing both EAEC 042 and UPEC-46 DNA; Lane 3, negative control using *E. coli* DH5α; Lane 4, negative control without DNA; Lanes 5–10, representative non-EAEC strains presenting the AA-pattern in adherence assays, previously classified as tEAEC, aEPEC, or STEC by molecular analysis. (**C**) Lane 1, 1 kb Plus DNA Ladder (Invitrogen); Lane 2, positive control - pool containing both EAEC 042 and UPEC-46 DNA; Lane 3, negative control using *E. coli* DH5α; Lane 4, negative control without DNA; Lanes 5-20, representative *aatA*+ EAEC strains.

After individual validation of selected primers, using respective control strains, a triplex-PCR protocol targeting *aggR, aatA*, and *afpR* was standardized by assessing different *E. coli* strains. EAEC, EPEC, and uropathogenic *E. coli* (UPEC) prototype strains, as well as *E. coli* DH5α, were tested. Figure 4A shows that only EAEC (042 and 17-2) and hybrid EAEC/UPEC (UPEC-46) presented amplicons corresponding to the targeted genes. In parallel, 24 *E. coli* strains genetically not classified as EAEC but exhibiting the AA pattern were also tested (Fig. 4B). As expected, all of them were negative for the presence of *aatA*, *aggR*, and *afpR*, supporting the specificity of this protocol.

Finally, the 163 clinical EAEC strains were tested using the triplex-PCR (Fig. 4C and Table 2). Detection of *aggR* using the universal primers in nine *aatA*+ strains previously considered *aggR*- (Table 2) shows an improvement in *aggR* detection, supporting the effectiveness and specificity of our molecular method for detecting EAEC strains. Regarding the detection of *aatA*, the results successfully reproduced previous data (12, 13), since 93.7% of these strains were positive. While 10 *aatA*-positive strains presented inconclusive results in our triplex-PCR, *aatA* amplification was clearly detected when tested in single reactions using the same primers (Fig. S4). All 23 strains previously detected as *aatA*+/*afpR*+ yielded consistent results with the new protocol (Table 2), confirming the efficacy of the new *afpR* primers.

**TABLE 2:** Comparison among *aggR*, *aatA*, and *afpR* positivity in the 163 EAEC strains using single or triplex-PCR

Genes	Percentage of positive results by PCR	
	Single PCR detection^*a*^	Triplex-PCR detection^*b*^
*aggR*	131 (80.4)	140 (85.9)
*aatA*	163 (100)	153 (93.9)^*c*^
*afpR*	23 (14.1)	23 (14.1)

^*a*^ Previously published PCR results are described in Table S2.

^*b*^ Results of triplex-PCR performed in the present study.

^*c*^ Ten negative strains were confirmed as *aatA*+ using the same primers used in the triplex-PCR, but in single reactions.

### The triplex-PCR results evidenced the presence of two distinct EAEC populations based on the presence or absence of *aggR*

The frequency of different EAEC-related genetic markers in the 163 EAEC strains was also assessed. Two main subgroups were clearly defined in our collection (Table 3): one subgroup consisting of 140 tEAEC (*aatA*+/*aggR*+/*afpR*-) that may or may not carry one of the AAF fimbriae; and the other comprising 23 aEAEC (*aatA*+/*aggR*-/*afpR*+), carrying the AFP operon.

**TABLE 3:** Occurrence of *aggR*, *aatA*, *afpR*, and EAEC adhesin-related genes (AAF, AFP and CS22) among 163 clinical EAEC strains

EAEC strains	Adhesin	Pilin gene	No. of strains (%)
Typical EAEC *aatA+/ aggR*+ /*afpR*- (*n* = 140)	AAF/I	*aggA+*	40 (28.6)
	AAF/V	*agg5A+*	22 (15.7)
	AAF/II	*aafA+*	18 (12.9)
	AAF/III	*agg3A+*	17 (12.1)
	AAF/IV	*agg4A+*	14 (10.0)
	AAF/III-V	*agg3A+/agg5A+*	5 (3.6)
	CS22	*cseA+*	19 (13.6)
	None	None	5 (3.6)
Atypical EAEC *aatA+/aggR*-/*afpR*+ (*n* = 23)	AFP	*afpA1+*	22 (95.7)
		*afpA1-*	1 (4.3)

## DISCUSSION

This study analyzed the primers currently used to detect typical EAEC-related genes (*aggR* and *aatA*) and *afpR* as a marker for atypical EAEC. Phenotypical and genotypical characterization of EAEC is either time-consuming or of variable sensitivity. Therefore, developing an accurate molecular diagnostic method for this *E. coli* pathotype is relevant for public health (30).

Nine different primers for *aggR* detection have been reported in the literature (Fig. 1), but a detailed analysis of their annealing sites has never been provided. In fact, our study is the first to demonstrate the existence of *aggR* sequence variants, suggesting that, over the years, many *E. coli* strains were probably not identified as EAEC in assays based on *aggR* detection with these primers, using in-house or commercial molecular kits. The comparative *in silico* analysis of *aggR* detection using each primer pair studied supports this hypothesis, as the frequency of *aggR* detection in our database ranged from 58% to 92.9%. Despite addressing these discrepancies, our analysis led to the proposal of a universal primer pair for *aggR* detection that completely anneals to conserved *aggR* regions, allowing the detection of all *aggR* variants based on all complete gene sequences available in public databases until November 2022 (Fig. 1).

Data generated from recent extensive genetic screenings and phylogenetic analysis (6, 14, 17, 26) highlighted two important facts about the EAEC pathotype. First, the evidence suggests that *aatA* is a specific genetic marker for the entire group, since there are no reports of the presence of *aatA* in other diarrheagenic *E. coli* pathotypes. Our analysis of the 24 pathogenic *E. coli* strains exhibiting the AA pattern in adherence assays (previously classified as EPEC or STEC) did not reveal any *aatA*+ strain. This gene is present in both pAA and pAFP plasmids, which are known to carry AA-related genetic markers, such as the AAF- and AFP-encoding operons, respectively (21, 24). Altogether, this evidence supports the specificity of *aatA* as a molecular marker for EAEC. Second, the existence of two main groups within the EAEC pathotype is suggested: tEAEC (*aatA*+/*aggR*+/*afpR*-) and aEAEC (*aatA*+/*aggR*-/*afpR*+) strains. Consequently, *afpR* is likely to be a specific genetic marker for aEAEC (17, 26).

EAEC diagnosis is currently a major challenge for clinical microbiologists due to the genotypic diversity of these strains. Likewise, some phenotypic characteristics that initially defined EAEC, such as the AA pattern, have also been observed in other *E. coli* pathotypes and even among other Enterobacterales, such as *Proteus mirabilis*, *Klebsiella pneumoniae*, and *Citrobacter freundii* (4, 31–33). Therefore, a rapid and effective molecular method is required for diagnosing this relevant diarrhea-causing agent (1, 30, 34).

Various molecular targets have been used to detect EAEC in epidemiological studies (1, 30), and a recent proposal suggested defining EAEC based on the presence of *aggR* along with either a complete AAF operon or the CS22 operon (35). Detecting all the genes that comprise the five AAF-encoding operons and the CS22 operon represents a significant challenge for molecular diagnostics. Also, the frequency of AAF-encoding genes varies among different EAEC collections (6, 14, 17, 26), and the specificity of *cseA* as a marker for EAEC detection is uncertain, given that it encodes the structural subunit of the fibrillar adhesin CS22, which was initially identified as a colonization factor of enterotoxigenic *E. coli* (36). Furthermore, this proposal fails to include aEAEC detection, as this group characteristically lacks *aggR*. The detection of atypical EAEC (aEAEC) is relevant, considering its isolation from sporadic acute diarrhea cases (17, 26), its involvement in significant diarrheal outbreaks among children and newborns (1, 4, 37, 38), and its demonstrated virulence in the *Galleria mellonella* infection model (39).

More recently, the AFP operon was characterized and linked to the AA phenotype in EAEC hybrid strains (24, 25), but its frequency among different EAEC strains has been poorly explored until now. Thus, clinically, distinguishing between typical and atypical EAEC strains is especially relevant for effective epidemiological surveillance and detection of hybrid strains, which may carry additional virulence factors and pose diagnostic and therapeutic challenges.

The first genetic tool used for EAEC detection was the CVD432 probe developed by Baudry et al. (15), which was further characterized as corresponding to *aatA* and detected using specific primers (23). Our triplex-PCR protocol, including these *aatA* primers, failed to amplify this target in 6.3% of the clinical EAEC strains previously selected based on this gene detection (12, 13). The lack of amplification in a triplex reaction may be due to the concentration of multiple primers, but a false-negative result for *aatA* does not compromise the diagnostic outcome. One strain would still be identified as EAEC with a negative result for *aatA*, but with amplification of *aggR* (*aatA*-/*aggR*+/*afpR*-) or *afpR* (*aatA*-/*aggR*-/*afpR*+). Another situation considering a false-negative *aatA* (*aatA*-/*aggR*-/*afpR*-) represents an EAEC genetic profile that has not been found in any of the genomes analyzed in this study.

In addition to detecting 100% of the *aggR* sequences in the database, the proposed universal primers improved detection in nine *aatA*-positive strains from our collection that had previously tested negative for *aggR* (Table 2). Concerning *afpR* detection (24), more specific primers were designed and validated, allowing a successful amplification in all 23 previously known *aatA*+/*afpR*+strains.

Finally, the extensive genetic characterization of our large EAEC collection (Table 3) clearly indicates two distinct groups of EAEC, also observed by others (6, 14, 17, 26): the tEAEC (*aatA+*/*aggR+*/AAF+ or *aatA+*/*aggR+*/*cseA+*) and the aEAEC (*aatA*+/*afpR*+/*afpA1*+) groups. It is interesting to mention the absence of pilin-related genes in 5 out of the 140 tEAEC and in one out of the 23 aEAEC, despite the presence of the respective regulators *aggR* and *afpR*, suggesting the presence of incomplete operons, pilin-encoding gene variants, or even uncharacterized adhesins in our collection.

In summary, the present study combined the use of universal *aggR* primers with primers targeting *aatA* and *afpR* and developed a triplex-PCR assay, which detects both typical and atypical EAEC and improves EAEC diagnosis in clinical laboratory routine.

## Data Availability

The authors confirm that the data supporting the findings of this study are available within the article and its supplementary materials. The raw data used in our analyzes are available in the Butantan Institute Repository (https://repositorio.butantan.gov.br/handle/butantan/13765).

## SUPPLEMENTAL MATERIAL

Figure S1 spectrum.01441-25-s0001.tif Segment representing one of the many *aatA* gene regions containing extensive adenine and thymine repetitions. Alignment of 251 *aatA* sequences downloaded from GenBank, using the MUSCLE algorithm available at the Geneious software (version 2025.0.2).

Figure S2 spectrum.01441-25-s0002.tif Agarose gel (0.8%) electrophoresis of *aggR* amplification using the universal primers selected in this study and BLASTn suite-2sequences analyze.

Figure S3 spectrum.01441-25-s0003.tif Agarose gel (0.8%) electrophoresis of *afpR* amplification using the universal primers selected in this study.

Figure S4 spectrum.01441-25-s0004.tif Reevaluation of the 10 EAEC strains considered inconclusive for *aatA* amplification by the triplex-PCR.

Table S1 spectrum.01441-25-s0005.pdf *E. coli* strains used as control in the standardization and validation of the triplex-PCR.

Table S2 spectrum.01441-25-s0006.pdf *E. coli* strains used to standardize and validate the triplex-PCR protocol.

Table S3 spectrum.01441-25-s0007.pdf Primers, amplification conditions, and control strains used in PCR screening of the major pilin-encoding genes for AAF/I to AAF/V, AFP, and CS22.

ASM does not own the copyrights to Supplemental Material that may be linked to, or accessed through, an article. The authors have granted ASM a non-exclusive, world-wide license to publish the Supplemental Material files. Please contact the corresponding author directly for reuse.
