# Supplementary figures and images for: Importance of aggR sequence variants detection for accurate molecular diagnosis of enteroaggregative Escherichia coli
Source: Microbiol Spectr. 2025 Sep 24;13(11):e01441-25. doi: 10.1128/spectrum.01441-25 (PMC12584630; doi:10.1128/spectrum.01441-25)

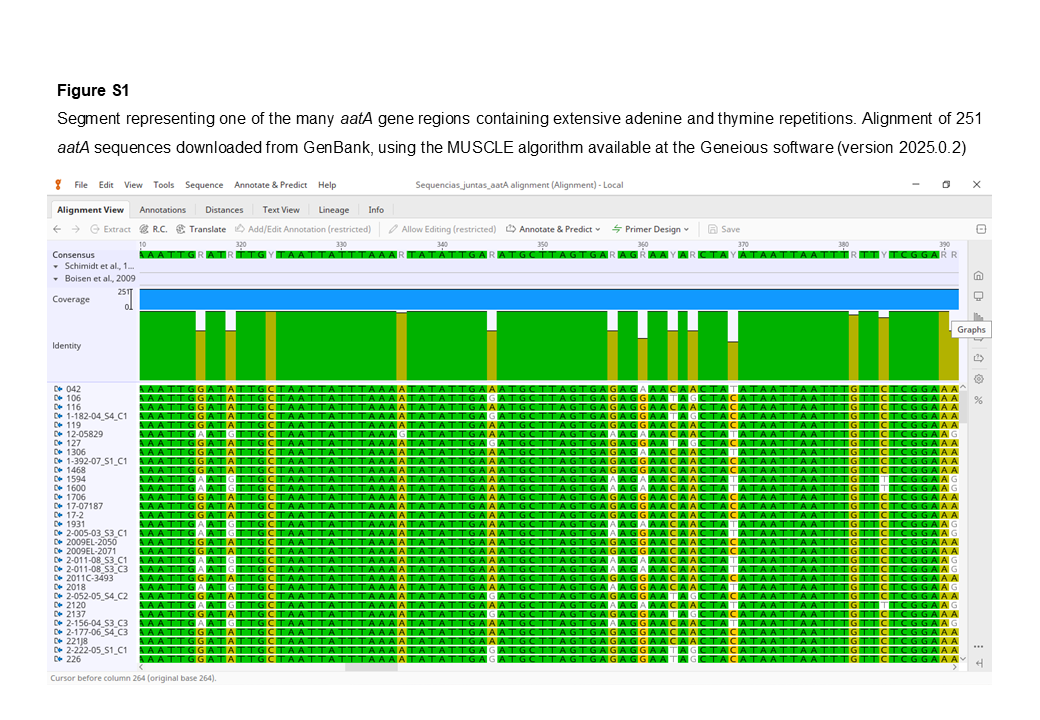

Supplement: Figure S1 — Segment representing one of the many aatA gene regions containing extensive adenine and thymine repetitions. Alignment of 251 aatA sequences downloaded from GenBank, using the MUSCLE algorithm available at the Geneious software (version 2025.0.2). [file spectrum.01441-25-s0001.tif]

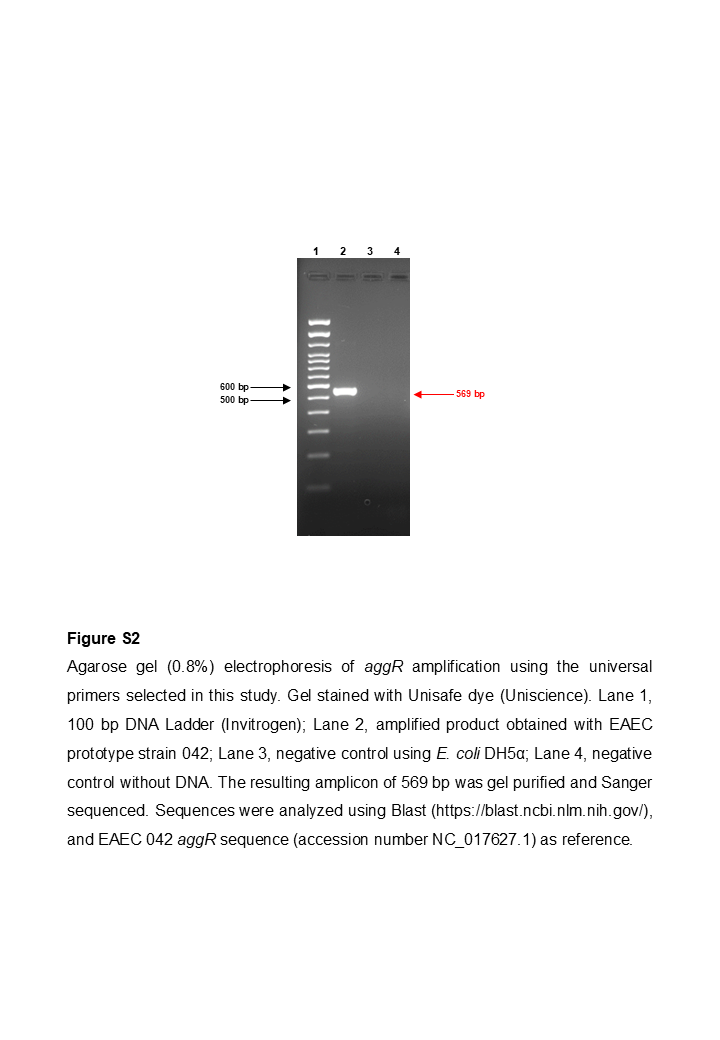

Supplement: Figure S2 — Agarose gel (0.8%) electrophoresis of aggR amplification using the universal primers selected in this study and BLASTn suite-2sequences analyze. [file spectrum.01441-25-s0002.tif]

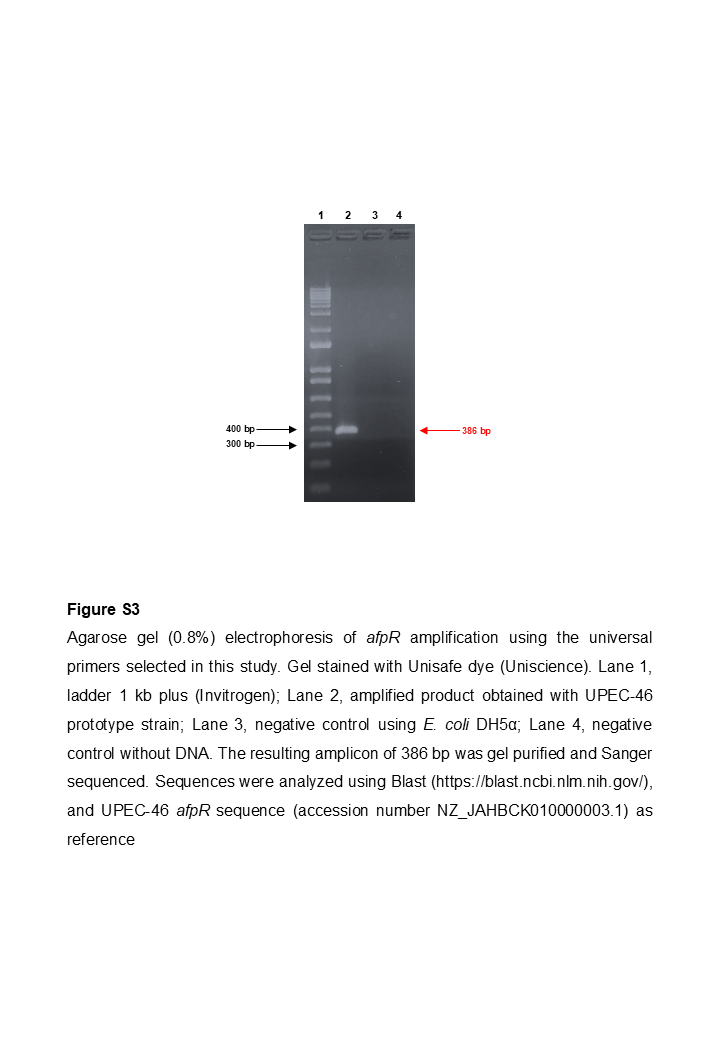

Supplement: Figure S3 — Agarose gel (0.8%) electrophoresis of afpR amplification using the universal primers selected in this study. [file spectrum.01441-25-s0003.tif]

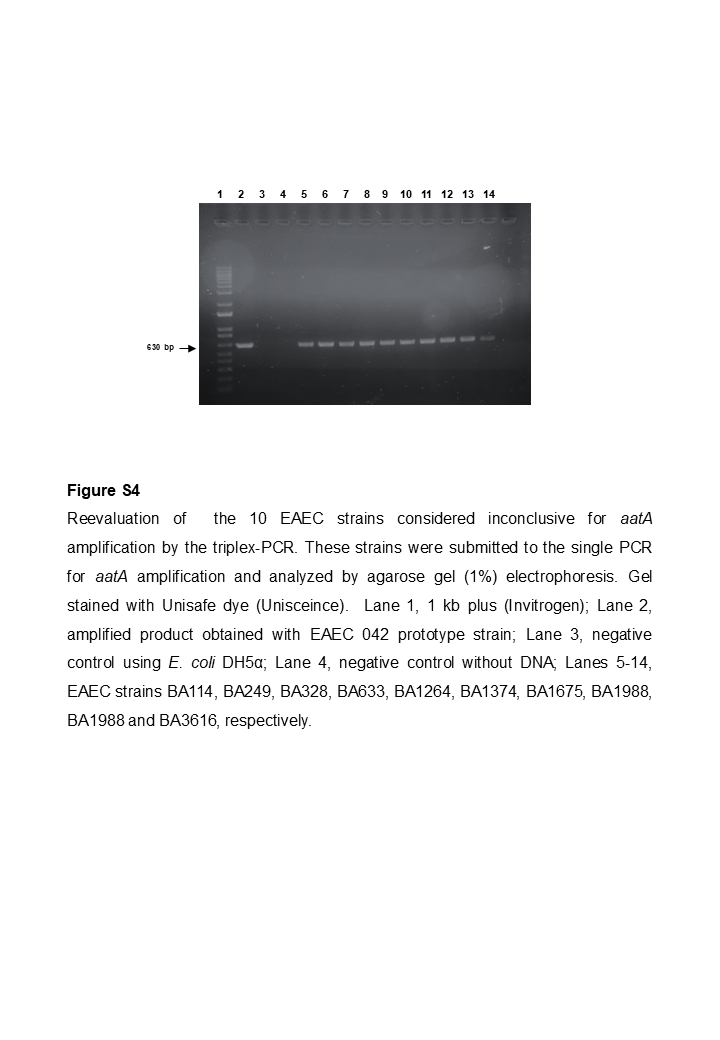

Supplement: Figure S4 — Reevaluation of the 10 EAEC strains considered inconclusive for aatA amplification by the triplex-PCR. [file spectrum.01441-25-s0004.tif]
