# Supplementary material for: Importance of aggR sequence variants detection for accurate molecular diagnosis of enteroaggregative Escherichia coli
Source: Microbiol Spectr. 2025 Sep 24;13(11):e01441-25. doi: 10.1128/spectrum.01441-25 (PMC12584630; doi:10.1128/spectrum.01441-25)
Supplement: Table S1 — E. coli strains used as control in the standardization and validation of the triplex-PCR. [file spectrum.01441-25-s0005.pdf]

Table S1

E. coli strains used as control in the standardization and validation of the triplex-PCR

Strain	***E. coli* pathotype**	EAEC genetic markers	References
042	typical enteroaggregative *E. coli* (tEAEC) prototype strain	***aatA+*** / ***aggR+*** / *afpR-*	(1)
17-2	typical enteroaggregative *E. coli* (tEAEC) prototype strain	***aatA+*** / ***aggR+*** / *afpR-*	(4)
UPEC-46	enteroaggregative *E. coli* (EAEC) isolated from urinary tract infection (EAEC/UPEC hybrid strain)	***aatA+*** / *aggR-* / ***afpR+***	(6)
E2348/69	typical enteropathogenic *E. coli* (tEPEC) prototype strain	*aatA-* / *aggR-* / *afpR-*	(2)
CFT073	uropathogenic *E. coli* (UPEC) prototype strain	*aatA-* / *aggR-* / *afpR-*	(3)
DH5α	*E. coli* K-12 strain	*aatA-* / *aggR-* / *afpR-*	(5)

References

1. Elias WP, Czeczulin JR, Henderson IR, Trabulsi LR, Nataro JP. 1999. Organization of biogenesis genes for aggregative adherence fimbria II defines a virulence gene cluster in enteroaggregative *Escherichia coli*. J Bacteriol 181:1779-1785. https://doi.org/10.1128/JB.181.6.1779-1785.1999.
2. Levine MM. 1987. *Escherichia coli* that cause diarrhea: enterotoxigenic, enteropathogenic, enteroinvasive, enterohemorrhagic, and enteroadherent. J Infect Dis 155:377–389. https://doi.org/10.1093/infdis/155.3.377.
3. Lloyd AL, Rasko DA, Mobley HLT. 2007. Defining genomic islands and uropathogen-specific genes in uropathogenic *Escherichia coli*. J Bact 189:3532–3546. https://doi.org/10.1128/JB.01744-06.
4. Nataro JP, Deng Y, Maneval DR, German AL, Martin WC, Levine MM. 1992. Aggregative adherence fimbriae I of enteroaggregative *Escherichia coli* mediate adherence to HEp-2 cells and hemagglutination of human erythrocytes. Infect Immun 60:2297-2304. https://doi.org/10.1128/iai.60.6.2297-2304.1992.
5. Sambrook J, Fritsch EF, Maniatis T. Molecular cloning: a laboratory manual. 2nd ed. Cold Spring Harbor, NY: Cold Spring Harbor Laboratory Press. 1989.
6. Schüroff PA, Salvador FA, Abe CM, Wami HT, Carvalho E, Hernandes RT, Dobrindt U, Gomes TAT, Elias WP. 2021. The aggregate-forming pili (AFP) mediates the aggregative adherence of a hybrid-pathogenic *Escherichia coli* (UPEC/EAEC) isolated from a urinary tract infection. Virulence 12:3073-3093. https://doi.org/10.1080/21505594.2021.2007645.
