# Supplementary material for: Importance of aggR sequence variants detection for accurate molecular diagnosis of enteroaggregative Escherichia coli
Source: Microbiol Spectr. 2025 Sep 24;13(11):e01441-25. doi: 10.1128/spectrum.01441-25 (PMC12584630; doi:10.1128/spectrum.01441-25)
Supplement: Table S2 — E. coli strains used to standardize and validate the triplex-PCR protocol. [file spectrum.01441-25-s0006.pdf]

Table S2

E. coli strains used to standardize and validate the triplex-PCR protocol

AA pattern in HEp-2 adherence assays		
No. of strains	Diarrheagenic *E. coli* pathotypes (serotypes)	References
24	typical EPEC (ONT:H45, O119:H11)	(1,3,6,7)
	atypical EPEC (ONT:H25, O2:H16, O86:H34, O105:H7, O111:H4, O111:H10, O111:H12, O111:H38, O125:H6, O128:H35, O157:H16)	
	Shiga toxin-producing *E. coli* (STEC): O93:H19 (1)	
**Clinical EAEC strains**		
No. of strains	EAEC-related genetic markers*	References
53	*aatA*; *aggR*; AAF/I-V pilin (major-subunit pilins); *cseA*; *afpR*; *afpA1*	(5)
110	*aatA*; *aggR*; AAF/I-V pilin (major-subunit pilins); *cseA*; *afpR*; *afpA1*	(2)

*EAEC-related markers listed have been investigated and published in previous studies [3,4], except for those underlined, which were investigated in the present study employing the methodology previously described [4].

References

1. Abe CM, Trabulsi LR, Blanco J, Blanco M, Dahbi G, Blanco JE, Mora A, Franzolin MR, Taddei CR, Martinez MB, Piazza RMF, Elias WP. 2009. Virulence features of atypical enteropathogenic *Escherichia coli* identified by the *eae*+ EAF-negative *stx*− genetic profile. Diag Microbiol Infect Dis 64:357–65. https://doi.org/10.1016/j.diagmicrobio.2009. 03.025.
2. Bueris V, Sircili MP, Taddei CR, dos Santos MF, Franzolin MR, Martinez MB, Ferrer SR, Barreto ML, Trabulsi LR. 2007. Detection of diarrheagenic *Escherichia coli* from children with

and without diarrhea in Salvador, Bahia, Brazil. Mem Inst Oswaldo Cruz 102:839-44. https://doi.org/10.1590/s0074-02762007005000116.

3. Elias WP, Barros SF, Moreira CG, Trabulsi LR, Gomes TA. 2002. Enteroaggregative *Escherichia coli* strains among classical enteropathogenic *Escherichia coli* O serogroups. J Clin Microbiol 40:3540-41. https://doi.org/10.1128/JCM.40.9.3540-3541.2002.
4. Freire CA, Rodrigues BO, Elias WP, Abe CM. 2022. Adhesin related genes as potential markers for the enteroaggregative *Escherichia coli* category. Front Cell Infect Microbiol 12:997208. https://doi.org/10.3389/fcimb.2022.997208.
5. Gomes TA, Vieira MA, Abe CM, Rodrigues D, Griffin PM, Ramos SR. 1998. Adherence patterns and adherence-related DNA sequences in *Escherichia coli* isolates from children with and without diarrhea in São Paulo city, Brazil. J Clin Microbiol 36:3609-13. https://doi.org/10.1128/JCM.36.12.3609-3613.1998.
6. Mairena EC, Neves BC, Trabulsi LR, Elias WP. 2004. Detection of LEE 4 region-encoded genes from different enteropathogenic and enterohemorrhagic *Escherichia coli* serotypes. Curr Microbiol 48:412-18. https://doi.org/10.1007/s00284-003-4164-8.
7. Vaz TM, Irino K, Nishimura LS, Cergole-Novella MC, Guth BE. 2006. Genetic heterogeneity of Shiga toxin-producing *Escherichia coli* strains isolated in Sao Paulo, Brazil, from 1976 through 2003, as revealed by pulsed-field gel electrophoresis. J Clin Microbiol 44:798-804. https://doi.org/10.1128/JCM.44.3.798-804.2006.
