# Supplementary material for: Importance of aggR sequence variants detection for accurate molecular diagnosis of enteroaggregative Escherichia coli
Source: Microbiol Spectr. 2025 Sep 24;13(11):e01441-25. doi: 10.1128/spectrum.01441-25 (PMC12584630; doi:10.1128/spectrum.01441-25)
Supplement: Table S3 — Primers, amplification conditions, and control strains used in PCR screening of the major pilin-encoding genes for AAF/I to AAF/V, AFP, and CS22. [file spectrum.01441-25-s0007.pdf]

Table S3

Primers, amplification conditions, and control strains used in PCR screening of the major pilin-encoding genes for AAF/I to AAF/V, AFP, and CS22

Genes	Oligonucleotides (5´-3´)	Amplicon (bp)	Annealing temp. (˚C)	Positive controls [a]	Ref.
aggA	(F) TCTATCTRGGGGGGCTAACG<br>(R) ACCTGTTCCCCATAACCAGAC	218	60	*aggA*: EAEC 17-2<br>*aafA*: EAEC 042<br>*agg3A*: EAEC RN785-1<br>*agg4A* and *agg3/4C*: EAEC BA1116 (GenBank accession number: ON920916)	1
aafA	(F) CTACTTTATTATCAAGTGGAGCCGCTA<br>(R) TAGGAGAGGCCAGAGTGWATCC	292			
agg3A	(F) AGCTAGTGCTACTGCAAAATTAAAGTT<br>(R) CAGGTTTAATATATTGGTCTGGAATAAC	359			
agg4a	(F) TGAGTTGTGGGGCTAYCTGGA<br>(R) CACCATAAGCCGCCAAATAAGC	169			
agg3/4C	(F) CATARTGAAGGTATAACATTTGGTCAGA<br>(R) GTCAGCATAACACTTACTGTTCATTC<br>(R) GTAGTTTGCATAGCAATGGCTATTCATT	477			
agg5A	(F) GTTTCATCAACTGGAACTATTACTATTT<br>(R) TAATTTAAGCTGAAGAATCCAGTCAAT	401	57	EAEC BA120 (GenBank accession number: ON920918)	1
afpA1	(F) AGAAGCGTAAAAGCTCCCTCC<br>(R) ACGGTGCTCTGAGTCTTGTT	140	55	UPEC-46	2
cseA	(F) CGCAAATGCCGCAACTGTA<br>(R) GCGTCTGGCAAATTCCAAC	348	55	EAEC BA249 (GenBank accession number: ON920917)	3

References:

1. Jønsson, R., Struve, C., Boll, E.J., Boisen, N., Joensen, K.G., Sørensen, C.A., et al. (2017b). A novel pAA virulence plasmid encoding toxins and two distinct variants of the fimbriae of enteroaggregative *Escherichia coli*. *Front. Microbiol.* 8, 1–12. doi: 10.3389/fmicb.2017.00263.

2. Schüroff, P.A., Salvador, F.A., Abe, C.M., Wami, H.T., Carvalho, E., Hernandes, R.T., et al. (2021). The aggregate-forming pili (AFP) mediates the aggregative adherence of a hybrid-pathogenic *Escherichia coli* (UPEC/EAEC) isolated from a urinary tract infection. *Virulence.* 12, 3073–3093. doi: 10.1080/21505594.2021.2007645.

3. Freire C.A., Rodrigues B.O., Elias W.P., Abe C.M. (2022). Adhesin related genes as potential markers for the enteroaggregative *Escherichia coli* category. *Front. Cell. Infect. Microbiol.* 12:997208. doi: 10.3389/fcimb.2022.997208.
